# Supplementary material for: Individual frailty excess hazard models in cancer epidemiology
Source: Stat Med. 2023 Jan 24;42(7):1066–81. doi: 10.1002/sim.9657 (PMC10560131; doi:10.1002/sim.9657)
Supplement: Supplementary file 1 — Appendix S1: Supporting Information [file SIM-42-1066-s001.pdf]

Received: Added at production | Revised: Added at production | Accepted: Added at production

DOI: xxx/xxxx

RESEARCH ARTICLE

Supplementary material: Individual frailty excess hazard models in cancer epidemiology

Francisco J. Rubio*[1] | Hein Putter[2] | Aurélien Belot[3]

[1]Department of Statistical Science, University College London, London, UK
[2]Department of Biomedical Data Sciences, Leiden University Medical Center, Leiden, The Netherlands
[3]Inequalities in Cancer Outcomes Network, Department of Non-Communicable Disease Epidemiology, London School of Hygiene & Tropical Medicine, London, UK

Correspondence
*Francisco J. Rubio, Department of Statistical Science, University College London, UK. Email: f.j.rubio@ucl.ac.uk

Funding information
...

1 | TECHNICAL DETAILS

Power Generalised Weibull distribution

The pdf, survival function, and hazard functions of the PGW are given below:

$$
\begin{aligned}
f(t;\sigma,\nu,\gamma) &= \frac{\nu}{\gamma\sigma^{\nu}} t^{\nu-1}\left[1+\left(\frac{t}{\sigma}\right)^{\nu}\right]^{\left(\frac{1}{\gamma}-1\right)} \exp\left\{1-\left[1+\left(\frac{t}{\sigma}\right)^{\nu}\right]^{\frac{1}{\gamma}}\right\},\\
S(t;\sigma,\nu,\gamma) &= \exp\left\{1-\left[1+\left(\frac{t}{\sigma}\right)^{\nu}\right]^{\frac{1}{\gamma}}\right\},\\
h(t;\sigma,\nu,\gamma) &= \frac{\nu}{\gamma\sigma^{\nu}} t^{\nu-1}\left[1+\left(\frac{t}{\sigma}\right)^{\nu}\right]^{\left(\frac{1}{\gamma}-1\right)},
\end{aligned}
$$

where $\sigma > 0$ is a scale parameter, and $\nu, \gamma > 0$ are shape parameters.

Calculation of (4)

The survival function $\tilde{S}_o(t;\mathbf{x})$ can be obtained by integrating out λ as follows,

$$
\tilde{S}_o(t;\mathbf{x}) = \int_0^{\infty} S_o(t \mid \lambda;\mathbf{x})dG(\lambda),
$$

where $S_o(t \mid \lambda; \mathbf{x}) = \exp\left\{-\int_0^t h_o(s \mid \lambda; \mathbf{x}) ds\right\}$. By using the decomposition (2), we can rewrite the conditional cumulative hazard as follows:

$$\tilde{H}_o(t \mid \lambda; \mathbf{x}) = \lambda H_E(t; \mathbf{x}) + H_P(\text{age} + t; \text{year} + t; \mathbf{z}) - H_P(\text{age}; \text{year}; \mathbf{z}).$$

Consequently,

$$\begin{aligned}\tilde{S}_o(t; \mathbf{x}) &= \int_0^\infty \exp\left\{-\left[H_P(\text{age} + t; \text{year} + t; \mathbf{z}) - H_P(\text{age}; \text{year}; \mathbf{z})\right] - \lambda H_E(t; \mathbf{x})\right\} dG(\lambda) \\ &= \exp\left\{-\left[H_P(\text{age} + t; \text{year} + t; \mathbf{z}) - H_P(\text{age}; \text{year}; \mathbf{z})\right]\right\} \int_0^\infty \exp\left\{-\lambda H_E(t; \mathbf{x})\right\} dG(\lambda),\end{aligned}$$

The second factor corresponds to the Laplace transform of G, evaluated at $H_E(t; \mathbf{x})$.

Gamma Individual Frailty Model

The Gamma distribution with unit mean and scale parameter $b > 0$ has Laplace transform:

$$\mathcal{L}_G(s; b) = \frac{1}{(1 + bs)^{\frac{1}{b}}}.$$

The derivative of this Laplace transform is:

$$\mathcal{L}'_G(s; b) = -\frac{1}{(1 + bs)^{\frac{1+b}{b}}}.$$

Consequently,

$$\frac{\mathcal{L}'_G(s; b)}{\mathcal{L}_G(s; b)} = -\frac{1}{(1 + bs)}.$$

Inverse Gaussian (IG) Individual Frailty Model

The IG distribution with unit mean and scale parameter $b > 0$ has Laplace transform:

$$\mathcal{L}_G(s; b) = \exp\left[\frac{1}{b}\left(1 - \sqrt{1 + 2sb}\right)\right].$$

The derivative of this Laplace transform is:

$$\mathcal{L}'_G(s; b) = -\frac{\exp\left[\frac{1}{b}\left(1 - \sqrt{1 + 2sb}\right)\right]}{\sqrt{1 + 2sb}}$$

Consequently,

$$\frac{\mathcal{L}'_G(s; b)}{\mathcal{L}_G(s; b)} = -\frac{1}{\sqrt{1 + 2sb}}$$

PVF Individual Frailty Model

The PVF distribution has Laplace transform [1]

$$\mathcal{L}_G(s; \rho, \nu, m) = \exp\left[-\rho\left\{1 - \left(\frac{\nu}{\nu + s}\right)^m\right\}\right],$$

with $\nu > 0$, $m > -1$, and $m\rho > 0$. The expectation and variance of a random variable S with PVF distribution are

$$\begin{aligned}E(S) &= \frac{\rho m}{\nu}, \\ \text{Var}(S) &= \frac{\rho m}{\nu}\frac{m + 1}{\nu}.\end{aligned}$$

Thus, in order to avoid non-identifiability issues in the context of individual net survival frailty models, it is necessary to impose the condition $\frac{\rho m}{\nu} = 1$. This can be achieved, for instance, by fixing $\nu = m\rho$. In such case, we have that the derivative of this Laplace transform is:

$$\mathcal{L}'_G(s;b) = -\exp\left\{-\rho\left(1-\left(\frac{m\rho}{m\rho+s}\right)^m\right)\right\}\left(\frac{m\rho}{m\rho+s}\right)^{m+1}.$$

Consequently,

$$\frac{\mathcal{L}'_G(s;b)}{\mathcal{L}_G(s;b)} = -\left(\frac{m\rho}{m\rho+s}\right)^{m+1}.$$

For a PVF frailty, the marginal individual survival function is given by

$$\begin{aligned}\tilde{S}_o(t;\mathbf{x}) &= \exp\{-H_P(\text{age}+t;\text{year}+t;\mathbf{z}) + H_P(\text{age};\text{year};\mathbf{z})\}\mathcal{L}_G\{H_E(t;\mathbf{x})\} \\ &= \exp\{-H_P(\text{age}+t;\text{year}+t;\mathbf{z}) + H_P(\text{age};\text{year};\mathbf{z})\}\exp\left[-\rho\left\{1+\left(\frac{\nu}{\nu+H_E(t;\mathbf{x})}\right)^m\right\}\right].\end{aligned}$$

Although the cumulative hazard and the hazard functions can be obtained in closed form, inference on these models tends to be more challenging.

Other tractable frailty distributions

Other tractable expressions can be obtained by replacing the expression for the Laplace transform associated to other distributions. In particular, the stable distribution derived in[5] and the Compound Poisson distribution proposed in[2] have tractable Laplace transforms which can be used instead of the PVF family of distributions discussed in the previous section. However, in order to allow for identifiability, as discussed in the following section, some additional restrictions need to be imposed on the parameter values which may complicate their implementation.

Identifiability

In this section, we show that a frailty survival model with GH structure and unit mean frailty is identifiable in the overall survival framework. We focus on the overall survival framework (this is, the case without expected mortality) as the identifiability of the frailty excess hazards model follows as a consequence of the identifiability of the corresponding frailty hazard model associated only to the excess hazard. Throughout, consider the following conditions and properties

A1. The random frailty λ is non-negative, with distribution G, and unit mean. This is, $E_G[\lambda] = 1$.

A2. Note that the cumulative distribution function of the GH model (associated only to the excess hazard) is

$$S_N(t;\mathbf{x}) = 1 - \mathcal{L}_G(H_E(t;\mathbf{x})) = 1 - E_\lambda\left[\exp\left\{\lambda H_E(t;\mathbf{x})\right\}\right],$$

where $\lambda \sim G$ and

$$H_E(t;\mathbf{x},\boldsymbol{\alpha},\boldsymbol{\beta},\boldsymbol{\theta}) = H_0\left(t\exp\{\mathbf{w}^\top\boldsymbol{\alpha}\};\boldsymbol{\theta}\right)\exp\left\{\mathbf{x}^\top\boldsymbol{\beta} - \mathbf{w}^\top\boldsymbol{\alpha}\right\}.$$

Thus, we can rewrite $H_E(t;\mathbf{x},\boldsymbol{\alpha},\boldsymbol{\beta},\boldsymbol{\theta}) = \phi(\mathbf{x})z(t,\mathbf{x})$, where $\phi(\mathbf{x}) = \exp\left\{\mathbf{x}^\top\boldsymbol{\beta} - \mathbf{w}^\top\boldsymbol{\alpha}\right\}$, and $z(t,\mathbf{x}) = H_0\left(t\exp\{\mathbf{w}^\top\boldsymbol{\alpha}\};\boldsymbol{\theta}\right)$. Consequently

$$\begin{aligned}S_N(t;\mathbf{x}) &= 1 - E_\lambda\left[\exp\left\{-\lambda\phi(\mathbf{x})z(t,\mathbf{x})\right\}\right] \\ &= 1 - \mathcal{L}_G\left(\phi(\mathbf{x})z(t,\mathbf{x})\right).\end{aligned}$$

Then,

$$z(t,\mathbf{x}) = \int_0^t h_0\left(u\exp\{\mathbf{w}^\top\boldsymbol{\alpha}\};\boldsymbol{\theta}\right)\exp\left\{\mathbf{x}^\top\boldsymbol{\alpha}\right\}du,$$

A3. We assume that $\mathbf{x} \in O$, where O is an open set in $\mathbb{R}^p$. This, together with the smoothness of the functions in the GH structure implies that the function $\phi(\mathbf{x})$ is non-negative, differentiable, and non-constant.

A4. We assume that the baseline hazard h_0 is not the hazard function of a Weibull distribution.

Under the above conditions and properties, it follows that Assumptions A1–A3 in[4] are satisfied. Then, as a consequence of the Theorem in[4], and the identifiability of the baseline GH model under Assumption A4[3,7], it follows that the proposed frailty GH model defined by (9) is identifiable.

Interpretation of the frailty

From equations (2) – (4), and assuming that G has a pdf g, we obtain:

$$\tilde{H}_o(t;\mathbf{x}) = [H_P(\text{age}+t;\text{year}+t;\mathbf{z}) - H_P(\text{age};\text{year};\mathbf{z})] - \log \int_0^{\infty} \exp\left[-\lambda H_E(t;\mathbf{x})\right] g(\lambda) d\lambda,$$

Differentiating with respect to t,

$$\begin{aligned}\tilde{h}_o(t;\mathbf{x}) &= h_P(\text{age}+t;\text{year}+t;\mathbf{z}) + \frac{\int_0^\infty \lambda h_E(t;\mathbf{x}) \exp\left[-\lambda H_E(t;\mathbf{x})\right] g(\lambda) d\lambda}{\int_0^\infty \exp\left[-\lambda H_E(t;\mathbf{x})\right] g(\lambda) d\lambda} \\ &= h_P(\text{age}+t;\text{year}+t,\mathbf{z}) - \frac{\mathcal{L}'_G\{H_E(t;\mathbf{x})\}}{\mathcal{L}_G\{H_E(t;\mathbf{x})\}} h_E(t;\mathbf{x}),\end{aligned}$$

where $\mathcal{L}'_G\{u\} = \frac{\partial}{\partial z}\mathcal{L}_G\{z\}\Big|_{z=u}$.

Similar to[6], the conditional distribution of the frailty λ among patients who survived beyond time t is

$$\begin{aligned} g(\lambda \mid T_o \geq t) &= \frac{P(T_o \geq t \mid \lambda) g(\lambda)}{P(T_o \geq t)} \\ &= \frac{S_o(t \mid \mathbf{x}, \lambda) g(\lambda)}{\tilde{S}_o(t;\mathbf{x})} \\ &= \frac{\exp\left[-\lambda H_E(t;\mathbf{x})\right] g(\lambda)}{\int_0^\infty \exp\left[-\lambda H_E(t;\mathbf{x})\right] g(\lambda) d\lambda} \\ &= \frac{S_N(t \mid \mathbf{x}, \lambda) g(\lambda)}{\tilde{S}_N(t;\mathbf{x})} \\ &= g(\lambda \mid T_C \geq t), \end{aligned}$$

which is not affected by the background mortality hazard. Then, the expected frailty of survivors can be written as

$$\begin{aligned} E[\lambda \mid T_o \geq t] &= \int_0^\infty \lambda g(\lambda \mid T_o \geq t) d\lambda \\ &= \frac{\int_0^\infty \lambda \exp\left[-\lambda H_E(t;\mathbf{x})\right] g(\lambda) d\lambda}{\int_0^\infty \exp\left[-\lambda H_E(t;\mathbf{x})\right] g(\lambda) d\lambda} \\ &= E[\lambda \mid T_C \geq t], \end{aligned}$$

where $T_o = \min\left\{T_P, T_C\right\}$, T_o is the survival time, T_P is the time to death from other causes, and T_C is the time to death from cancer. Consequently,

$$\tilde{h}_o(t;\mathbf{x}) = h_P(\text{age}+t;\text{year}+t;\mathbf{z}) + h_E(t;\mathbf{x}) E[\lambda \mid T_o \geq t].$$

This implies that the marginal hazard function can be decomposed as the sum of the population hazard and the excess hazard at time t multiplied by the mean of the frailty associated to the survivors beyond time t.

2 | SIMULATION RESULTS: AIM 1

Parameter	True	MeanMLE	Bias	MedianMLE	Coverage	Mean StdErr	EmpSD
**Scenario N=500**							
σ	0.750	0.854	0.104	0.729	0.829	0.400	0.475
ν	1.500	1.608	0.108	1.591	0.951	0.190	0.196
γ	5.000	5.776	0.776	5.265	0.838	2.637	3.011
α_1	0.700	0.584	-0.116	0.698	0.967	4.160	1.106
α_2	0.700	0.806	0.106	0.704	0.933	0.304	0.479
β_1	1.000	1.083	0.083	1.009	0.970	2.043	0.633
β_2	0.500	0.422	-0.078	0.486	0.979	0.165	0.297
β_3	0.500	0.531	0.031	0.516	0.936	0.201	0.217
β_4	0.750	0.791	0.041	0.751	0.920	0.224	0.247
b	1.000	1.156	0.156	0.977	0.893	0.698	0.873
**Scenario N=1000**							
σ	0.750	0.844	0.094	0.765	0.894	0.317	0.358
ν	1.500	1.550	0.050	1.534	0.947	0.122	0.126
γ	5.000	5.185	0.185	5.040	0.902	1.889	2.023
α_1	0.700	0.617	-0.083	0.695	0.968	1.382	0.801
α_2	0.700	0.758	0.058	0.706	0.961	0.205	0.320
β_1	1.000	1.053	0.053	1.017	0.964	0.682	0.395
β_2	0.500	0.461	-0.039	0.490	0.971	0.100	0.189
β_3	0.500	0.524	0.024	0.520	0.940	0.143	0.149
β_4	0.750	0.788	0.038	0.773	0.943	0.164	0.170
b	1.000	1.155	0.155	1.071	0.918	0.541	0.634
**Scenario N=2000**							
σ	0.750	0.769	0.019	0.717	0.919	0.216	0.242
ν	1.500	1.531	0.031	1.526	0.957	0.084	0.082
γ	5.000	5.280	0.280	5.272	0.916	1.398	1.440
α_1	0.700	0.682	-0.018	0.688	0.969	0.220	0.231
α_2	0.700	0.719	0.019	0.703	0.947	0.117	0.135
β_1	1.000	1.011	0.011	1.006	0.962	0.103	0.114
β_2	0.500	0.488	-0.012	0.496	0.970	0.052	0.062
β_3	0.500	0.504	0.004	0.499	0.944	0.099	0.098
β_4	0.750	0.759	0.009	0.751	0.947	0.113	0.111
b	1.000	1.038	0.038	0.984	0.945	0.380	0.424
**Scenario N=5000**							
σ	0.750	0.760	0.010	0.743	0.941	0.137	0.142
ν	1.500	1.512	0.012	1.509	0.949	0.051	0.050
γ	5.000	5.113	0.113	5.077	0.946	0.884	0.887
α_1	0.700	0.690	-0.010	0.695	0.964	0.132	0.133
α_2	0.700	0.713	0.013	0.708	0.965	0.068	0.069
β_1	1.000	1.008	0.008	1.006	0.955	0.059	0.059
β_2	0.500	0.495	-0.005	0.497	0.975	0.028	0.028
β_3	0.500	0.506	0.006	0.501	0.951	0.062	0.063
β_4	0.750	0.759	0.009	0.757	0.947	0.071	0.074
b	1.000	1.028	0.028	1.014	0.947	0.238	0.247

TABLE 1 Simulation results for Aim 1, scenario 2. MeanMLE: Mean of the Maximum Likelihood Estimates; MedianMLE: median value of the Maximum Likelihood Estimates; Mean StdErr: Mean of the standard errors; EmpSD: Empirical standard deviation

Parameter	True	MeanMLE	Bias	MedianMLE	Coverage	Mean StdErr	EmpSD
**Scenario N=500**							
σ	0.200	0.203	0.003	0.191	0.913	0.063	0.070
ν	1.500	1.582	0.082	1.560	0.950	0.175	0.183
γ	4.000	4.425	0.425	4.112	0.885	1.503	1.705
α_1	0.700	0.672	-0.028	0.680	0.966	0.419	0.445
α_2	-0.700	-0.752	-0.052	-0.705	0.932	0.315	0.372
β_1	1.000	1.014	0.014	1.006	0.966	0.205	0.208
β_2	0.500	0.547	0.047	0.524	0.960	0.178	0.199
β_3	0.500	0.510	0.010	0.509	0.946	0.189	0.191
β_4	-0.750	-0.772	-0.022	-0.757	0.946	0.206	0.214
b	1.000	1.069	0.069	1.021	0.918	0.479	0.537
**Scenario N=1000**							
σ	0.200	0.201	0.001	0.195	0.932	0.046	0.048
ν	1.500	1.538	0.038	1.525	0.950	0.114	0.118
γ	4.000	4.222	0.222	4.042	0.931	1.037	1.112
α_1	0.700	0.677	-0.023	0.694	0.954	0.278	0.285
α_2	-0.700	-0.730	-0.030	-0.710	0.936	0.215	0.229
β_1	1.000	1.007	0.007	1.006	0.955	0.134	0.138
β_2	0.500	0.514	0.014	0.507	0.955	0.117	0.123
β_3	0.500	0.510	0.010	0.507	0.952	0.131	0.134
β_4	-0.750	-0.764	-0.014	-0.757	0.942	0.144	0.150
b	1.000	1.039	0.039	1.021	0.955	0.342	0.359
**Scenario N=2000**							
σ	0.200	0.200	-0.000	0.197	0.949	0.033	0.032
ν	1.500	1.520	0.020	1.516	0.949	0.078	0.077
γ	4.000	4.121	0.121	4.056	0.949	0.726	0.694
α_1	0.700	0.700	0.000	0.698	0.952	0.192	0.199
α_2	-0.700	-0.702	-0.002	-0.698	0.955	0.150	0.150
β_1	1.000	1.004	0.004	1.001	0.955	0.092	0.089
β_2	0.500	0.509	0.009	0.506	0.957	0.080	0.079
β_3	0.500	0.506	0.006	0.504	0.963	0.091	0.088
β_4	-0.750	-0.758	-0.008	-0.756	0.951	0.101	0.101
b	1.000	1.013	0.013	1.000	0.942	0.240	0.239
**Scenario N=5000**							
σ	0.200	0.201	0.001	0.200	0.941	0.021	0.021
ν	1.500	1.509	0.009	1.507	0.942	0.048	0.048
γ	4.000	4.021	0.021	3.995	0.945	0.445	0.448
α_1	0.700	0.696	-0.004	0.700	0.955	0.121	0.119
α_2	-0.700	-0.711	-0.011	-0.708	0.948	0.094	0.094
β_1	1.000	1.004	0.004	1.002	0.941	0.058	0.058
β_2	0.500	0.505	0.005	0.505	0.947	0.050	0.053
β_3	0.500	0.504	0.004	0.503	0.947	0.058	0.058
β_4	-0.750	-0.758	-0.008	-0.756	0.946	0.064	0.064
b	1.000	1.025	0.025	1.019	0.937	0.152	0.156

TABLE 2 Simulation results for Aim 1, scenario 3. MeanMLE: Mean of the Maximum Likelihood Estimates; MedianMLE: median value of the Maximum Likelihood Estimates; Mean StdErr: Mean of the standard errors; EmpSD: Empirical standard deviation

3 | SIMULATION RESULTS: AIM 2

In this section, we present the simulation results discussed in Section 5 of the main paper concerning Aim 2. Figures 1 and 2 show the net survival curves associated to the two strategies described in the main paper.

In addition, we analyse the integrated absolute errors (IAE) obtained by calculating the integrals

$$\text{IAE} = d(S_1, S_2) = \int_0^{\tau} |S_1(t) - S_2(t)| dt,$$

where τ is an upper limit defined by the follow-up time of interest, and S_1 and S_2 are survival functions. We present two types of comparisons between the frailty model and the classical model. First, we compare the frailty model and the classical model

against the true generating model. That is, we calculate

$$\text{IAE} = d(\widehat{S}, S^*) = \int_0^\tau |\widehat{S}(t) - S^*(t)|dt,$$

where $\widehat{S}$ represents the corresponding fitted net survival (classical or frailty), and S^* represents the true net survival curve of interest. The IAE values illustrate the discrepancies between the true and the fitted net survival curves in the region of interest. Table 3 shows the IAEs for the frailty (F) *vs.* true net survival curves, and for the for the classical (C) *vs.* true net survival curves. We notice that the (*e.g.* mean and median) IAEs between the stratified models and the true generating models decreases as the sample size increases. On the other hand, the IAEs between non-stratified models remains high even for large samples. This indicates that the non-stratified inference is biased due to model misspecification (associated to IUH) in the corresponding subgroup of interest ($X_1 = 0, 1$), and that this bias does not decrease with increasing sample sizes.

In a second setting, we directly compare the frailty model against the classical model. That is, we calculate

$$\text{IAE} = d(\widehat{S}_F, \widehat{S}_C) = \int_0^\tau |\widehat{S}_F(t) - \widehat{S}_C(t)|dt,$$

where $\widehat{S}_F$ represents the fitted frailty net survival function, and $\widehat{S}_C$ represents the fitted classical net survival function. The IAE values illustrate the discrepancies between the fitted net survival curves, obtained with the two models, in the region of interest. Table 4 shows the IAEs for the frailty (F) *vs.* the classical (C) net survival curves. "Population" indicates the case where population net survival functions are compared, while the remaining cases represent the comparison of subgroups associated to $X_1 = 0, 1$, using the non-stratified (NS) and stratified (S) models. We can see that the pairs of fitted models are relatively close (in terms of the IAEs), but their (*e.g.* mean and median) IAEs do not decrease with increasing sample sizes.

We note that one could also calculate the integral over the entire $(0, \infty)$ as this is a finite quantity. However, this strategy will indicate discrepancies in regions that are not of interest in practice.

3.1 | Aim 2

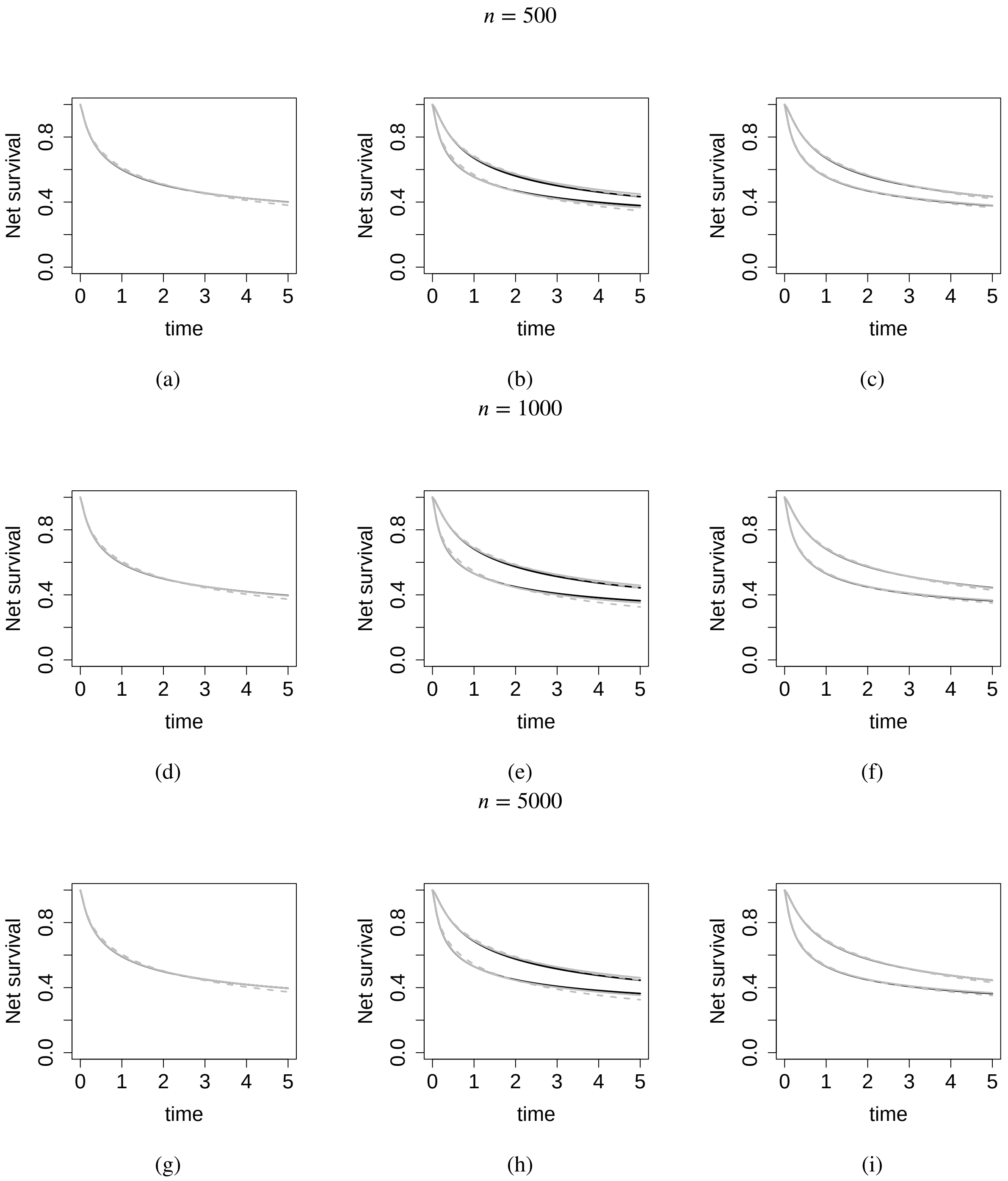

FIGURE 1 First scenario, Aim 2 (True model - black line, Frailty model - gray lines, Classical model- gray dashed lines): (a,d,g) net survival curves for the entire population, (b,e,h) net survival curves for each strata, (c,f,i) net survival curves for each strata based on a stratified analysis.

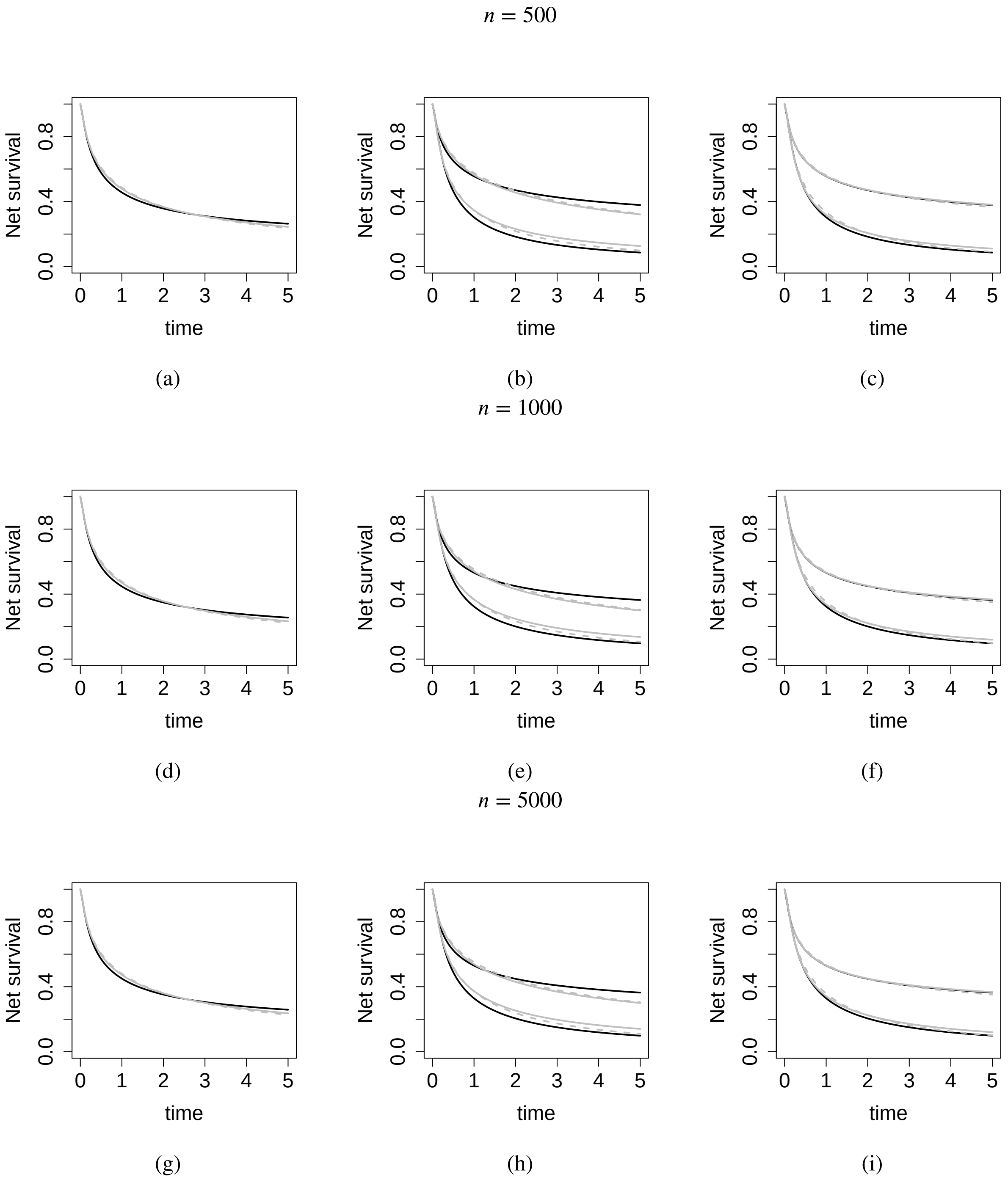

FIGURE 2 Second scenario, Aim 2 (True model - black line, Frailty model - gray lines, Classical model- gray dashed lines): (a,d,g) net survival curves for the entire population, (b,e,h) net survival curves for each strata, (c,f,i) net survival curves for each strata based on a stratified analysis.

	Min.	1st Qu.	Median	Mean	3rd Qu.	Max.
			$n = 500$			
Population F	0.020	0.081	0.112	0.129	0.165	0.412
Population C	0.041	0.097	0.123	0.139	0.167	0.415
$X_1 = 0$, NSF	0.010	0.129	0.204	0.214	0.297	0.594
$X_1 = 0$, SF	0.007	0.067	0.118	0.135	0.182	0.481
$X_1 = 0$, NSC	0.019	0.079	0.134	0.153	0.214	0.509
$X_1 = 0$, SC	0.015	0.067	0.108	0.129	0.173	0.470
$X_1 = 1$, NSF	0.041	0.121	0.174	0.197	0.253	0.601
$X_1 = 1$, SF	0.008	0.072	0.124	0.143	0.195	0.572
$X_1 = 1$, NSC	0.048	0.122	0.160	0.181	0.227	0.527
$X_1 = 1$, SC	0.006	0.070	0.123	0.142	0.194	0.514
			$n = 1000$			
Population F	0.023	0.073	0.094	0.102	0.118	0.359
Population C	0.048	0.094	0.109	0.118	0.131	0.355
$X_1 = 0$, NSF	0.014	0.139	0.197	0.203	0.259	0.613
$X_1 = 0$, SF	0.004	0.056	0.097	0.109	0.150	0.483
$X_1 = 0$, NSC	0.024	0.073	0.122	0.132	0.178	0.493
$X_1 = 0$, SC	0.005	0.055	0.086	0.101	0.134	0.452
$X_1 = 1$, NSF	0.063	0.128	0.167	0.184	0.226	0.589
$X_1 = 1$, SF	0.005	0.050	0.086	0.102	0.136	2.640
$X_1 = 1$, NSC	0.073	0.127	0.154	0.168	0.195	0.520
$X_1 = 1$, SC	0.012	0.051	0.086	0.100	0.135	0.451
			$n = 5000$			
Population F	0.043	0.065	0.072	0.073	0.080	0.148
Population C	0.069	0.090	0.096	0.096	0.102	0.145
$X_1 = 0$, NSF	0.086	0.178	0.208	0.208	0.236	0.345
$X_1 = 0$, SF	0.005	0.065	0.089	0.090	0.113	0.219
$X_1 = 0$, NSC	0.035	0.102	0.126	0.128	0.153	0.248
$X_1 = 0$, SC	0.017	0.054	0.075	0.078	0.100	0.203
$X_1 = 1$, NSF	0.098	0.140	0.160	0.164	0.185	0.294
$X_1 = 1$, SF	0.002	0.021	0.037	0.044	0.060	0.183
$X_1 = 1$, NSC	0.103	0.131	0.145	0.148	0.162	0.248
$X_1 = 1$, SC	0.012	0.031	0.043	0.050	0.063	0.176

TABLE 3 Second scenario, Aim 2: IAE for the frailty (F) *vs.* true net survival curves, and for the for the classical (C) *vs.* true net survival curves. "Population" indicates the case where population net survival functions are compared, while the remaining cases represent the comparison of subgroups associated to $X_1 = 0, 1$, using the non-stratified frailty (NSF), stratified frailty (SF), non-stratified classical (NSC), and stratified classical (SC) models.

	Min.	1st Qu.	Median	Mean	3rd Qu.	Max.
			$n = 500$			
Population	0.000	0.012	0.021	0.023	0.032	0.077
$X_1 = 0$, NS	0.000	0.054	0.080	0.080	0.105	0.257
$X_1 = 0$, S	0.000	0.050	0.063	0.062	0.073	0.134
$X_1 = 1$, NS	0.000	0.028	0.044	0.045	0.058	0.154
$X_1 = 1$, S	0.000	0.012	0.032	0.037	0.055	0.538
			$n = 1000$			
Population	0.000	0.018	0.025	0.025	0.032	0.064
$X_1 = 0$, NS	0.000	0.066	0.083	0.084	0.101	0.176
$X_1 = 0$, S	0.012	0.052	0.060	0.061	0.069	0.128
$X_1 = 1$, NS	0.000	0.036	0.045	0.047	0.057	0.098
$X_1 = 1$, S	0.000	0.015	0.036	0.040	0.056	2.637
			$n = 5000$			
Population	0.012	0.022	0.025	0.025	0.029	0.043
$X_1 = 0$, NS	0.046	0.077	0.086	0.087	0.095	0.129
$X_1 = 0$, S	0.041	0.055	0.059	0.059	0.062	0.081
$X_1 = 1$, NS	0.025	0.043	0.048	0.048	0.053	0.075
$X_1 = 1$, S	0.000	0.025	0.036	0.035	0.045	0.078

TABLE 4 Second scenario, Aim 2: IAE for the frailty (F) *vs.* the classical (C) net survival curves. “Population” indicates the case where population net survival functions are compared, while the remaining cases represent the comparison of subgroups associated to $X_1 = 0, 1$, using the non-stratified (NS) and stratified (S) models.

References

1. O. Aalen, O. Borgan, and H. Gjessing. *Survival and Event History Analysis: a Process Point of View*. Springer-Verlag, New York, 2008.

2. O.O. Aalen. Modelling heterogeneity in survival analysis by the compound Poisson distribution. *The Annals of Applied Probability*, 2(4):951–972, 1992.

3. Y.Q. Chen and N.P. Jewell. On a general class of semiparametric hazards regression models. *Biometrika*, 88(3):687–702, 2001.

4. C. Elbers and G. Ridder. True and spurious duration dependence: The identifiability of the proportional hazard model. *The Review of Economic Studies*, 49(3):403–409, 1982.

5. P. Hougaard. Survival models for heterogeneous populations derived from stable distributions. *Biometrika*, 73(2):387–396, 1986.

6. P. Hougaard. Frailty models for survival data. *Lifetime Data Analysis*, 1(3):255–273, 1995.

7. F.J. Rubio, L. Remontet, N.P. Jewell, and A. Belot. On a general structure for hazard-based regression models: an application to population-based cancer research. *Statistical Methods in Medical Research*, 28:2404–2417, 2019.

□
